# High contiguity genome sequence of a multidrug-resistant hospital isolate of *Enterobacter hormaechei*

**DOI:** 10.1186/s13099-019-0288-7

**Published:** 2019-02-13

**Authors:** Leigh G. Monahan, Matthew Z. DeMaere, Max L. Cummins, Steven P. Djordjevic, Piklu Roy Chowdhury, Aaron E. Darling

**Affiliations:** 1 ithree institute, University of Technology Sydney, Broadway Street, Ultimo, 2007 Australia; 2 NSW Department of Primary Industries, Elizabeth Macarthur Agricultural Institute, Woodbridge Road, Menangle, 2568 Australia

**Keywords:** *Enterobacter hormaechei*, *Enterobacter cloacae* complex, Long-read sequencing, Hybrid assembly

## Abstract

**Background:**

*Enterobacter hormaechei* is an important emerging pathogen and a key member of the highly diverse *Enterobacter cloacae* complex. *E. hormaechei* strains can persist and spread in nosocomial environments, and often exhibit resistance to multiple clinically important antibiotics. However, the genomic regions that harbour resistance determinants are typically highly repetitive and impossible to resolve with standard short-read sequencing technologies.

**Results:**

Here we used both short- and long-read methods to sequence the genome of a multidrug-resistant hospital isolate (C15117), which we identified as *E. hormaechei*. Hybrid assembly generated a complete circular chromosome of 4,739,272 bp and a fully resolved plasmid of 339,920 bp containing several antibiotic resistance genes. The strain also harboured a 34,857 bp repeat encoding copper resistance, which was present in both the chromosome and plasmid. Long reads that unambiguously spanned this repeat were required to resolve the chromosome and plasmid into separate replicons.

**Conclusion:**

This study provides important insights into the evolution and potential spread of antimicrobial resistance in a nosocomial *E. hormaechei* strain. More broadly, it further exemplifies the power of long-read sequencing technologies, particularly the Oxford Nanopore platform, for the characterisation of bacteria with complex resistance loci and large repeat elements.

## Background

The *Enterobacter cloacae* complex (ECC) is a diverse group of bacterial species of both clinical and environmental importance [1]. ECC bacteria are associated with a variety of different infections in humans and have emerged as one of the leading causes of nosocomial infections worldwide [2, 3]. Importantly, ECC strains are intrinsically resistant to a number of antibiotics and have demonstrated a remarkable ability to acquire additional resistance determinants. These can include extended spectrum beta-lactamases (ESBLs) and carbapenemases, in some cases severely limiting available treatment options [2].

Accurate identification of ECC isolates at the species level is important, particularly in the clinical setting where specific ECC subgroups are more likely to cause nosocomial infections or outbreaks [4]. However, this has proven difficult due to imprecise taxonomy and the failure of standard phenotypic tests to discriminate between ECC members. Successful identification of ECC species typically relies on DNA sequencing, and has been demonstrated using approaches such as *hsp60* typing [5] and phylogenomics [6].

Sequence-based analysis also plays a critical role in characterising antibiotic resistance in ECC isolates. However, this can be complicated by the fact that antibiotic regions are often flanked by repetitive insertion sequences and cannot be resolved by standard short read methods, leading to a loss of critical information on the structure and genomic context of resistance determinants [7].

In this study, we use a combination of short-read (Illumina) and long-read (Oxford Nanopore) technologies to sequence a multidrug-resistant, ESBL-positive strain of ECC isolated from the general environment of an Australian hospital (C15117). We identify this strain as *Enterobacter hormaechei*, which is one of the most prevalent causes of human infection among ECC members and a species that is increasingly recognised for its ability to persist and spread in hospital environments. Using a hybrid assembly approach, we demonstrate that C15117 harbours a large plasmid (340 kb) with multiple drug resistance determinants and we generate fully resolved assemblies of both the plasmid and genome. This was not possible with short read data alone, highlighting the utility of long read technologies for precise characterisation of extrachromosomal replicons in clinical isolate sequencing, which often play significant roles in the spread of antimicrobial resistance.

## Methods

### Strain isolation and antibiotic resistance profiling

Strain C15117 was isolated from the burns ward at Concord Repatriation Hospital in Sydney, Australia. MALDI-TOF analysis was used for initial bacterial identification, while antibiotic resistance profiling was performed using the automated VITKEK-2 system (bioMérieux) and further confirmed via synergy testing with plate assays. This revealed the isolate to be an ESBL-producing strain of ECC with resistance to ampicillin (MIC = 32 μg/ml), augmentin (32 μg/ml), ticarcillin/clavulanic acid (64 μg/ml), piperacillin/tazobactam (64 μg/ml), cefazolin (64 μg/ml), cefoxitin (64 μg/ml), ceftazidime (64 μg/ml), ceftriaxone (16 μg/ml), gentamycin (16 μg/ml), tobramycin (8 μg/ml), trimethoprim (16 μg/ml) and trimethoprim/sulfamethoxazole (320 μg/ml). Susceptibility breakpoints were as defined in the EUCAST breakpoint tables for interpretation of MICs and zone diameters (version 8.0; http://www.eucast.org).

### DNA preparation and quality control

DNA was isolated from C15117 using the xanthogenate-SDS (XS) extraction method of Tillet and Neilan [8] with several modifications. First, 6 ml of stationary phase culture was harvested by centrifugation and resuspended in 50 μl of TER buffer containing 200 μg/ml RNAse A. Cells were then resuspended in 1 ml of XS buffer and incubated at 50 °C for 2 h. After completing the remainder of the Tillet and Neilan [8] protocol, additional purification steps were performed. This involved first resuspending the sample in 500 μl of buffer B1 from the Blood and Cell Culture DNA Midi Kit (Qiagen) and incubating at 50 °C overnight. An additional 2 ml of buffer B1 was then added and the sample was further purified by following the kit protocol for Gram-negative bacterial DNA extraction from step 5 onwards (Qiagen), including treatment with proteinase K but omitting the addition of lysozyme and RNAse A.

DNA yield was measured using a Qubit 2.0 fluorometer (Thermo Scientific), while quality was assessed by agarose gel electrophoresis and Nanodrop (Thermo Scientific) spectrophotometry. This confirmed that the sample was of sufficient purity (A260 nm/A280 nm of 1.85; A260 nm/A230 nm of 2.06) and molecular weight (>40 kb with no small DNA contamination) for long-read sequencing without further purification or size selection.

### DNA sequencing

Illumina library preparation and sequencing were performed as described previously [9], except that 2 × 150 nt paired-end reads were generated using MiSeq V2 chemistry.

For long-read MinION sequencing, libraries were prepared using the 1D ligation sequencing kit (SQK-LSK108) from Oxford Nanopore Technologies (ONT) with several modifications to the standard ONT protocol. The optional shearing step was avoided to maximise read length, while to improve throughput the amount of starting DNA was increased to 10 μg (compared to 1 μg in the standard protocol; also see [7]). DNA purifications steps were performed using SPRIselect beads (Beckman Coulter), with bead resuspension carried out at higher than usual temperatures (50 °C after end repair and 37 °C after adapter ligation) to promote efficient elution of high molecular weight DNA into solution. The final library containing 3.75 μg of DNA was loaded onto an ONT MinION instrument with a FLO-MIN107 (R9.5) flow cell and run for 48 h as per the manufacturer’s instructions. Live base-calling was not performed during the run.

Single Molecule, Real-Time (SMRT) sequencing was conducted at the Ramaciotti Centre for Genomics at the University of New South Wales (Sydney, Australia) using a PacBio RSII instrument (Pacific Biosystems).

### Basecalling

After completion of the ONT MinION run, the resulting fast5 reads were base-called using the read_fast5_basecaller from the ONT Albacore Sequencing Pipeline Software (version 2.1.3) with command-line options “-r -k SQK-LSK108 -f FLO-MIN107”.

### Assembly and annotation

Two hybrid genome assemblies were generated, in each case combining the reads from the Illumina short-read library with one of the two long-read libraries (ONT, PacBio). The Unicycler assembly pipeline (version 0.4.3) [10] was employed with default command-line options for both assemblies.

Automated genome annotation was performed on the RAST annotation server, using the RAST-tk scheme [11]. The annotation can be accessed with guest login, under RAST ID 158836.149. Preliminary identification of antibiotic resistance genes and insertion sequences was performed using ResFinder 3.1 [12] and ISfinder [13], respectively. All predicted genes were thereafter confirmed by manual BLASTn and BLASTp searches.

### Phylogenetic analysis

**Fig. 1:**
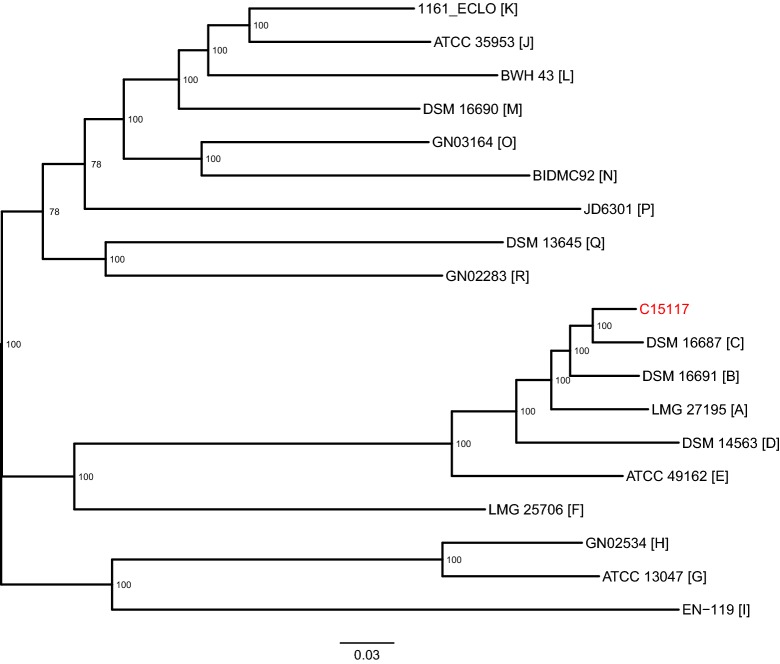
Phylogenetic analysis identifies C15117 as most closely related to *Enterobacter hormaechei* susbp. *oharae*. Representative strains from 18 ECC phylogenomic groups (labelled A to R in square brackets) were included in the analysis. Bootstrap support values are shown at the nodes of the tree. Branch length has been normalised to substitutions per site

To perform a phylogenetic analysis of C15117, representative genome sequences from each of the 18 ECC phylogenomic groups [6] were obtained from the PATRIC database [14]. From this set of 19 genome sequences, with C15117 as the alignment reference, a reference-based genome alignment was inferred using Snippy (version 4.3.6, https://github.com/tseemann/snippy) (4,739,272 columns). From the initial alignment, Mothur (version 1.41.0) [15] was used to remove any columns containing gaps or ambiguous bases, while retaining both variant and shared columns (1,043,814 columns). Next, areas of recombination were predicted using Gubbins (version 2.3.4) [16] under a GTRGAMMA model in RAxML (version 8.2.12) [17] (205,516 columns). After filtering, a phylogenetic tree with support values was inferred using RAxML under a GTRGAMMA model, with extended majority rule consensus and rapid bootstrapping (Fig. 1).

## Results and discussion

### Sequencing and assembly

We initially sequenced strain C15117 using short-read Illumina technology only (2 × 150 nt paired-end reads), generating 507,053 reads representing 76 Mbp (approximately 15-fold coverage). However, we found that the resulting genome assembly using SPAdes (version 3.11.1) remained highly fragmented (> 200 contigs) due to the presence of multiple repetitive insertion sequences, including 10 copies of IS*26*. Importantly, this meant that the genetic context of the major antibiotic resistance determinants could not be resolved.

To circumvent this problem, we settled on a hybrid approach in which Illumina short reads were combined with long reads generated on the ONT MinION platform and co-assembled using the software tool Unicycler [10]. Unlike Illumina data, which is highly accurate, Nanopore reads are known to contain systematic errors that cannot be fully eliminated by computing a consensus sequence, even when high depth of coverage is available [18]. By combining the two data types, Unicycler is capable of producing assemblies that are accurate in terms of both sequence and structure.

A 48 h MinION run generated 73,879 long reads (min: 193 bp, max: 202,611 bp, median: 8462 bp, mean: 15,888 bp) representing 1.73 Gbp. From an initial short-read SPAdes assembly, Unicycler fully aligned 61,410 and partially aligned 12,080 long-reads, with a mean alignment identity of 87.2% and totaling 1.167 × 10^9^ aligned bases. After polishing with Pilon (version 1.22) [19], the bridged assembly graph was composed of 4 components: a complete circular chromosome of 4,739,272 bp; a large fully resolved plasmid of 339,920 bp that we have designated pSPRC-Echo1 (NCBI BioProject Accession PRJNA494598); and two smaller plasmids of 6237 bp and 2496 bp. Basic assembly statistics are shown in Table 1. Genome annotation using RAST [11] identified a total of 4946 predicted coding sequences and 110 RNA genes.

**Table 1:** Assembly statistics from the ONT + Illumina hybrid assembly of C15117, calculated using Quast (version 2.3) [25]

Statistic	Value
No. contigs	7
No. contigs > 1 kb	6
Total length (bp)	5,096,894
Total length > 1 kb	5,096,275
Longest contig	4,739,272
GC (%)	54.99
N50	4,739,272
L50	1
Ns per 100 kb	0.00

### Species identification and phylogenetic analysis

Initial testing of C15117 using MALDI-TOF identified the strain only at the level of the *Enterobacter cloacae* complex. For species-level identification, phylogenetic analysis was performed using representative strains from each of the 18 phylogenomic groups that make up the ECC (A to R), as defined in a recent comprehensive study by Chavda and colleagues [6]. C15117 was found to be most closely related to phylogenomic group C type strain DSM 16687, identifying it as *Enterobacter hormaechei* susbp. *oharae* (Fig. 1). This also places the isolate in Hoffman cluster VI, one of 12 genetic clusters previously described for the ECC based on *hsp60* sequencing [5]. Multilocus sequence typing using PubMLST [20] showed the strain belongs to sequence type (ST) 61.

*Enterobacter hormaechei* is an important emerging pathogen, and the most frequently isolated ECC from human clinical specimens [1]. It has been reported in several outbreaks of sepsis, most notably in the USA and Brazil, while subsp. *oharae* specifically has been linked with infections of orthopaedic implants [21]. Critically, *E. hormaechei* has also been noted for its ability to persist in hospital environments, where it may act as a reservoir for infection and the transmission of antibiotic resistance [2, 4].

To identify publicly available genomes closely related to C15117, we utilised the “Similar Genome Finder” tool within the PATRIC database [14], which computes the distance between two given sequences via Mash [22]. Interestingly, the most closely related public genome was assembled from a metagenomic sample isolated from a metal surface in New York City (BioSample Accession SAMN06456256; Mash distance 0.00251, corresponding to an Average Nucleotide Identity of about 99.7%). Other closely related genomes include a collection of 16 strains isolated as part of an antibiotic resistance surveillance project from the Sanger Institute (BioProject PRJEB5065).

### Resistance genes

Multiple genes were identified in C15117 that are known to confer antibiotic resistance and likely contribute to the observed resistance profile of the organism (see "Methods"). These include two copies of *bla*_SHV-12_ encoding ESBL resistance, as well as several other β-lactamases and genes conferring resistance to aminoglycosides and sulfonamides. Interestingly, all of the clinically relevant resistance determinants in C15117 were located on plasmid pSPRC-Echo1, along with all 10 copies of IS*26*. This has important implications for understanding the evolution of resistance and its potential transmission from this strain, and could only be resolved with the use of ONT long-read data.

### Duplication of a copper resistance module

**Fig. 2:**
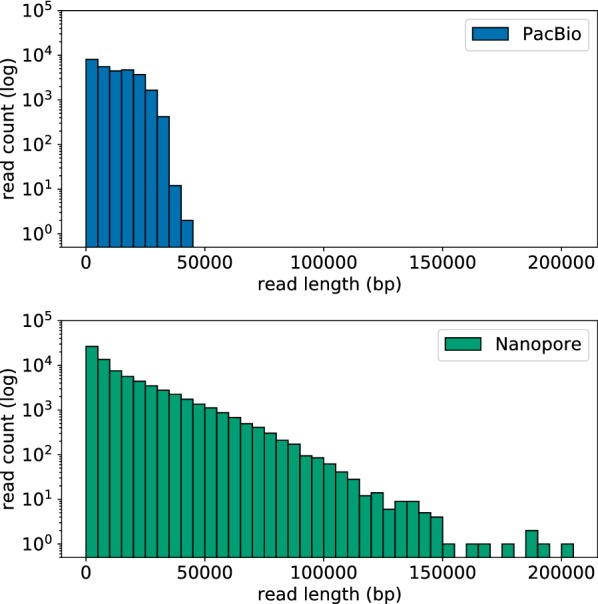
Distribution of read lengths generated on the PacBio RSII (top) and the ONT MinION (bottom) for *E. hormaechei* C15117. Maximum read lengths were 202,611 bp for ONT and 44,753 bp for PacBio; minima were 193 bp (ONT) and 35 bp (PacBio); means were 15,888 bp (ONT) and 11,881 bp (PacBio), while median lengths were 8462 bp (ONT) and 10,629 bp (PacBio)

Our initial attempts to resolve the pSPRC-Echo1 plasmid and C15117 chromosome into separate replicons used long-read data generated with an alternative technology (PacBio), but the approach failed. This was despite using the same hybrid assembly technique (with the same Illumina read set) as described above for ONT. By aligning both of the long-read data sets to the fully resolved ONT/Illumina hybrid genome, we found that the discrepancy between the two assemblies was likely due to the presence of a large repeat that is shared between the chromosome and plasmid. This 34,857 bp repeat, present both in the chromosome and the plasmid, encodes a copper resistance module flanked by Tn7-like transposons. Multiple ONT reads were found to unambiguously span the repeat on the chromosome (45 reads) and plasmid (30 reads). Conversely, no PacBio reads completely spanned the repeat on either chromosome or plasmid. This disparity between the two data sets is unlikely to be the result of differences in sequence depth or uniformity of coverage, but rather a striking difference in the distribution of read lengths generated by the two technologies (see Fig. 2). Very few PacBio reads exceeded the repeat length of 35 kb, while ONT produced a high proportion of such reads up to a maximum of 203 kb.

Although it is certainly possible to generate PacBio reads of sufficient length to resolve a 35 kb repeat, the ONT platform appears to be inherently better suited to this kind of analysis. ONT read lengths are limited only by the physical length of the fragment to be sequenced, meaning that with careful DNA extraction and library processing it is possible to generate single reads in excess of 1 Mb [23]. In contrast, maximum read lengths with PacBio technology are inherently limited by the sequencing chemistry itself [24]. As exemplified here, the study of antibiotic resistant bacteria with complex, unpredictable genome structures is one area in which the importance of read length is clear.

## Abbreviations

ECC: *Enterobacter cloacae* complex
ESBL: extended spectrum beta-lactamase
MALDI-TOF: matrix assisted laser desorption/ionisation time-of-flight mass spectrometry
MIC: minimum inhibitory concentration
EUCAST: European Committee on Antimicrobial Susceptibility Testing
XS: xanthogenate-SDS
TER: Tris-EDTA-RNAse A buffer
ONT: Oxford Nanopore Technologies
SMRT: single molecule, real-time sequencing
SNV: single nucleotide variant
RAST: Rapid Annotation using Subsystem Technology
ST: sequence type
PATRIC: Pathosystems Resource Integration Center
